# Impact of home-based training and nutritional behavior on body composition and metabolic markers in cancer patients: data from the CRBP-TS study

**DOI:** 10.3389/fnut.2023.1152218

**Published:** 2023-09-19

**Authors:** Sophia Darmochwal, Christian Bischoff, René Thieme, Ines Gockel, Uwe Tegtbur, Peter Hillemanns, Antina Schulze, Johannes Voss, Roberto Falz, Martin Busse

**Affiliations:** ^1^Institute of Sport Medicine and Prevention, University Leipzig, Leipzig, Germany; ^2^Department of Visceral, Transplant, Thoracic and Vascular Surgery, University Hospital Leipzig, Leipzig, Germany; ^3^Department of Rehabilitation and Sports Medicine, Hannover Medical School, Hannover, Germany; ^4^Department of Gynecology and Obstetrics, Hannover Medical School, Hannover, Germany

**Keywords:** cancer, body composition, online-based home training, metabolic markers, Adiponectin, Leptin

## Abstract

**Introduction:**

Obesity and physical inactivity are known to affect cancer's development and prognosis. In this context, physical aerobic and resistance training as well as a Mediterranean nutrition have been proven to have many positive health effects. The aim of this study was therefore to investigate the effect of home-based training on body composition and certain metabolic laboratory parameters.

**Methods:**

Patients with breast, colorectal and prostate cancer who underwent curative surgery at stages T1N0M0–T3N3M0 were eligible for this trial and randomized to an intervention and control group. In the intervention group the patients carried out online-based strength-endurance home training during the 6-month study period. Body composition was assessed via bioelectrical impedance analysis (baseline, 3 months and 6 months). Metabolic blood parameters were also analyzed and nutrition behavior determined using the Mediterranean Diet Adherence Screener (MEDAS).

**Results:**

The intervention group's fat mass decreased while their lean body mass increased (time effect *p* = 0.001 and *p* = 0.001, respectively). We found no interaction effect in body weight (*p* = 0.19), fat mass [*p* = 0.06, 6-months estimates −0.9 (95% CI −1.8 to −0.1)] and lean body mass (*p* = 0.92). Blood samples also failed to show a statistically significant interaction effect between time × group for HbA1c% (*p* = 0.64), Insulin (*p* = 0.33), Adiponectin (*p* = 0.87), Leptin (*p* = 0.52) and Triglycerides (*p* = 0.43). Only Adiponectin revealed significance in the time effect (*p* < 0.001) and Leptin in the group effect (*p* = 0.03). Dietary behavior during the study period was similar in patients in the intervention and control groups (interaction *p* = 0.81; group *p* = 0.09 and time *p* = 0.03).

**Discussion:**

Individualized online-based home training in postoperative cancer patients revealed only minor changes, with no group differences in body composition or metabolic laboratory parameters, which were predominantly in the reference range at baseline. More studies investigating effects of online-based home training on body composition and nutrition behavior are needed.

**Trial registration:**

https://drks.de/search/en/trial/DRKS00020499, DRKS-ID: DRKS00020499.

## 1. Introduction

Among women worldwide, most develop breast cancer (24.5%), colorectal cancer (9.4%) and lung cancer (8.4%), whereas men develop lung cancer ranked first (14.3%), followed by prostate cancer (14.1%) and colorectal cancer (10.6%) (1).

Compelling evidence indicates that physical activity improves cancer-related health outcomes, and that physical exercise is generally safe during cancer therapy (2). Exercise and physical activity are beneficial for cancer patients at all stages (3–5) for helping to prevent various types of cancer and for surviving cancer overall (6–12), as it alleviates fatigue, loss of strength, diabetes mellitus, and metabolic syndrome while enhancing endurance performance and quality of life (13–15). Moreover, regular exercise reduces the risks of recurrence (16), recurrence mortality, and overall mortality in breast cancer and prostate patients (12, 17, 18). Individualized training, namely its frequency, intensity, and the patient's pre-and postoperative physical condition are important here (19, 20). The current physical activity guidelines recommend 150–300 min per week of moderate (3–5.9 METs) or an equivalent amount of vigorous intensity aerobic activity of 75–150 min per week (<6 METs). Online-based home training has thus become increasingly popular in recent years. The first studies have shown that home-based training also lowers body weight, body fat mass, and fasting insulin levels, and raises adiponectin levels (21, 22). However, the effects of distance-based exercise interventions on physical capacity and body composition reported so far have been small (23). Essential factors that help patients maintain their adherence to a training program include considering their individual capacity, giving them motivation-enhancing activity feedback, and bidirectional communication (24). Telemedicine-based exercise interventions in cancer patients enable measured activity tracking, but actual physical activity is usually self-reported (23).

Being overweight is one of the main factors contributing to cancer's development (25–27). Excess weight also triggers deviations in metabolic laboratory markers such as Adiponectin, Leptin, Triglycerides, and fasting Insulin (27).

A well-studied way to counteract obesity is the Mediterranean diet (MD) with its proven positive health effects (28). An MD effectively reduces body weight as well as risk factors for metabolic syndrome (29, 30). Cancer-positive impacts of MD have also been observed (31–33). A protective effect for gastric (34), colorectal (35), and bladder cancer (36) is particularly evident. A valid tool for assessing the MD is the Mediterranean Diet Adherence Screener (MEDAS) questionnaire, which asks about its implementation via 14 questions (37, 38).

Considering the insufficient evidence on the effects of home-based training and nutrition on body composition and metabolic markers, this paper's aim was to test whether online training and Mediterranean nutritional behavior would result in changes in body composition and certain metabolic laboratory markers in breast, prostate, and colorectal cancer patients.

## 2. Materials and methods

### 2.1. Study design and study population

ColoRectal, Breast, and Prostate Cancer-Telemonitoring and Self-management (CRBP-TS) was a prospective, multicenter randomized, controlled parallel-group trial done as a collaborative project conducted by Leipzig University (Institute of Sport Medicine and Prevention and Department of Visceral, Transplant, Thoracic and Vascular Surgery, University Hospital of Leipzig), the Hannover Medical School, and University Hospital Dresden (Germany). The study was approved by the Ethics Committee of the Medical Faculty, University of Leipzig (reference number 056/20-ek) and at all participating sites. The present analysis focuses on the body composition and metabolic data from the “CRBP-TS” study. Our primary and secondary study endpoints (cardiopulmonary exercise testing, physical activity data and, safety assessment) are described in Falz et al. (39).

Written informed consent was obtained from all participants. Eligible subjects were female and male cancer patients with International Classification of Diseases codes C18/19/20 (colorectal cancer), C50 (breast cancer), and C61 (prostate cancer) who underwent curative (R0) surgery at stages T1N0M0 to T3N3M0. Further inclusion criteria were an age between 18 and 75, Eastern Co-operative Oncology Group (ECOG) <1 without acute cardiac, renal, hepatic, endocrine, bone marrow or cerebral disorder and the cognitive ability to understand the postoperative program and participate actively. Of our screened patients, 148 were included in the study at three study sites in Germany.

### 2.2. Study plan, measurements and data collection

After recruitment, all patients were randomly assigned (1:1 allocation; stratified by study site and cancer entity; Clinical Trial Center Leipzig) to the intervention group (IG) or control group (CG). The study and data collection period lasted 6 months. Data collection occurred at baseline (T1), at 3 months (T2) and at 6 months (T3). All participants underwent an incremental exertion test at T1, T2, and T3, and further testing. A detailed description of the study design has been published (39, 40). Additional end points of changes in flow-mediated dilatation, blood parameters (inflammation panel, tumor makers, miRNAs) and questionnaires (Patient Health Questionnaire-2, Depression Anxiety Stress Scale, Fatigue Severity Scale and Oral Health Impact Profile) from baseline to 6 months are not reported here.

#### 2.2.1. Online-based home training and CRBP-TS application

The IG participated in individual online-based home training involving strength and endurance exercises with the instruction to train accordance with exercise guidelines (2, 41) for two (at least) or preferably three times or more, with counseling as needed. The strength endurance exercises mainly done with the patient's own body weight included for example stepping exercises, squats, rowing, upper body push and pull exercises, jumps and core exercises. The target training intensity was determined by the perceived exertion (target 5–8; CR10 scale) and by relying on an individual maximum heart rate (75% heart rate max or symptom-limited heart rate) defined during the cardiopulmonary exercise test at baseline.

All study participants were given a tablet (Lenovo Tab M10 TB-X606X; Lenovo, Hongkong, China) and an activity device (Vivoactive 4; Garmin, Olathe, Kansas, US) for activity tracking. The CRBP-TS application (Diavention GmbH, Leipzig, Germany) was installed on the tablet and the activity device was connected to the tablet via Bluetooth. The CRBP-TS app was used to visualize training videos and transfer the heartrate data via chest belt from the device throughout the training, to receive activity feedback (steps per day, activity time), and to fill in different questionnaires. The app of the CG was not equipped with training videos. The CG received standard care and basic information on lifestyle changes and physical activity according to the guidelines, as well as the wearable to get information on their activity (steps per day, activity time).

#### 2.2.2. Clinical assessments

Body height and weight were measured to calculate the body mass index. Segmental bioimpedance served to analyze fat mass and lean body mass (*Lean body mass* is defined as the difference between total body weight and fat mass) (Leipzig & Dresden: BIACORPUS RX 4004M, MEDI CAL HealthCare GmbH, Germany; Hannover: InBody720; Biospace, Seoul, Republic of Korea). Thereby, a low-level electric current flows through the body tissue. Varying resistance to the current flow is shown depending on the type of tissue. Fatty tissue triggers strong impedance and structures, whereas tissue with aqueous content reveals low impedance (42).

Blood samples were collected at T1, T2, and T3 to assess potentially predictive outcome factors. The adipose and metabolic markers used in this study included HbA1c, Leptin, Adiponectin, Insulin, and Triglycerides. Ethylenediaminetetraacetic acid plasma was analyzed at each study center and serum was collected, centrifuged, and stored at −80°C until analysis. All serum samples were analyzed in a central core laboratory (Institute of Laboratory Medicine, Clinical Chemistry and Molecular Diagnostics, University Hospital Leipzig).

The Mediterranean Diet Adherence Screener (MEDAS) was used to determine patients' adherence to the Mediterranean diet. It includes 14 questions in all, 12 questions on food consumption and frequencies, and 2 questions on eating habits considered to characterize MD (43). The evaluation is done by earning 0 or 1 point (Table 1). If a criterion is not met, 0 points are recorded.

**Table 1:** MEDAS questionnaire (38).

**Questions**	**Criteria for 1 point**
1. Do you use olive oil as main culinary fat?	Yes
2. How much olive oil do you consume in a given day (including oil used for frying, salads, out-of-house meals, etc.)?	≥4 tbsp
3. How many vegetable servings do you consume per day? [1 serving: 200 g (consider side dishes as half a serving)]	≥2 (≥1 portion raw o raw a salad)
4. How many fruit units (including natural fruit juices) do you consume per day?	≥3
5. How many servings of red meat, hamburger, or meat products (ham, sausage, etc.) do you consume per day? (1 serving: 100–150 g)	<1
6. How many servings of butter, margarine, or cream do you consume per day? (1 serving: 12 g)	<1
7. How many sweet or carbonated beverages do you drink per day?	<1
8. How much wine do you drink per week?	≥7 glasses
9. How many servings of legumes do you consume per week? (1 serving: 150 g)	≥3
10. How many servings of fish or shellfish do you consume per week? (1 serving 100–150 g of fish or 4–5 units or 200 g of shellfish)	≥3
11. How many times per week do you consume commercial sweets or pastries (not homemade), such as cakes, cookies, biscuits, or custard?	<3
12. How many servings of nuts (including peanuts) do you consume per week? (1 serving: 30 g)	≥3
13. Do you preferentially consume chicken, turkey, or rabbit meat instead of veal, pork, hamburger, or sausage?	Yes
14. How many times per week do you consume vegetables, pasta, rice, or other dishes seasoned with sofrito (sauce made with tomato and onion, leek, or garlic and simmered with olive oil)?	≥2

### 2.3. Statistical analyses

All values are expressed as the means and standard deviation unless otherwise stated, and the significance level was defined as *p* < 0.05. Data were analyzed via IBM SPSS Statistics (Version 29; IBM, Armonk, New York, USA) and displayed using GraphPad Prism (Version 9; GraphPad Software Inc., California, USA). For distribution analysis, the D'Agostino-Pearson normality test was used. The evaluation was conducted on an intention-to-treat basis, and all randomized participants were included. Per-protocol analyses were conducted including only IG participants who completed all study visits and who had engaged in at least 1.5 training sessions per week. All analyses were two-sided, and the level of significance was *p* = 0.05. To evaluate the endpoints, we applied mixed-effects models with a repeated-measurements structure (estimated using restricted maximum likelihood). In this model, the measured values (baseline, 3-month and 6-month follow-ups) were treated as the dependent variable. As fixed effects we have included the randomization arm and categorical time covariate in the model. Interactions were modeled for group and time. As random effect(s), an intercept for subjects was used. Within the mixed models, we calculated 95% confidence intervals and *p*-values for contrasts between groups for the 3- and 6-month periods. In a sensitivity analysis, only those patients were included who had complete paired baseline and 6-month follow-ups for time difference within groups (paired *t* test for dependent samples).

## 3. Results

Table 2 illustrates the entire sample's baseline clinical characteristics (*N* = 148) by randomized group assignment. The groups showed baseline imbalances.

**Table 2:** Baseline characteristics in the intervention vs. control group.

	**Mean (SD)**	**Mean (SD)**
	**Intervention group (*****n*** = **76)**	**Control group (*****n*** = **72)**
Age (years)	54.4 ± 1	54.6 ± 1
**Sex**		
Female (%)	45 (59)	43 (60)
Male (%)	31 (41)	29 (40)
Height (cm)	172 ± 8.2	171 ± 11.0
**Body composition**		
Weight (kg)	78.8 ± 15.3	74.9 ± 15.3
Fat mass (kg)	24.6 ± 10.3	21.3 ± 8.4
Lean body mass (kg)	54.2 ± 11.1	53.6 ± 10.6
Body mass index (kg/m^2^)	26.9 ± 4.5	25.1 ± 4.7
Waist to hip ratio	0.90 ± 0.2	0.88 ± 0.1
**Cancer entity no. (%)**		
Colorectal cancer	10 (13)	9 (12)
Breast cancer	43 (57)	41 (57)
Prostate cancer	23 (30)	22 (31)
Dropouts no. (%)	14 (18)	12 (17)
SAE's no. (hospitalizations)	11	7
**Comorbidities no (%)**		
Diabetes type 2	4 (5)	2 (3)
Hypertension	23 (30)	17 (24)
Adipositas	4 (5)	0 (0)
Cardiovascular diseases	2 (3)	3 (4)
Hypothyroidism	13 (17)	15 (21)
Asthma	2 (3)	2 (3)

Values are presented as the means and standard deviation.

All results of the mixed model for body composition, laboratory markers, MEDAS, physical activity parameters are in Table 3 (intent-to-treat analysis). Sixty-two patients completed the 6 months study period in IG (14 dropouts). Fourty-six (74%) of those patients performed at least 1.5 training sessions per week. Results of the per-protocol analysis were similar to the main results of the trial (Supplementary Table S1) and three-month visit data are presented in Supplementary Table S2.

**Table 3:** Laboratory parameters, body composition, nutrition questionnaire and activity score at baseline and after 6 months (intent to treat analysis; mixed model with repeated measurements; T1–T3).

	**Mean (SD) [sample size]**						**Difference^b^ 6 months IG vs. CG (95% CI)**	**Time effect^c^**	**Group effect^c^**	**Inter-action effect^c^**
	**Intervention group**			**Control group**				***p*-value**	***p*-value**	**Group × time *p*-value**
	**T1**	**T3**	**Diff^a^**	**T1**	**T3**	**Diff^a^**				
**Laboratory**										
HbA1c%	5.4 (0.4) [76]	5.5 (0.3) [62]	0.04 (0.4) [62]	5.4 (0.6) [71]	5.4 (0.3) [59]	0.05 (0.3) [59]	−0.017 (−0.15 to 0.09)	0.18	0.71	0.64
Insulin (pmol/L)	68.8 (53.2) [76]	62.9 (32.4) [61]	−2.3 (34.9) [61]	63.9 (53.5) [71]	61.8 (38.9) [59]	−1.1 (49.5) [59]	7.2 (−18 to 33)	0.81	0.71	0.33
Adiponectin (mg/L)	8.4 (5.6) [76]	9.5 (7.6) [62]	1.4^*^ (3.9) [62]	9.1 (5.5) [71]	10.1 (5.8) [59]	1.3^*^ (3.3) [59]	0.2 (−0.8 to 1.3)	**<0.001**	0.49	0.87
Leptin (ng/mL)	17.0 (20.3) [75]	14.8 (15.8) [62]	−2.2 (10.8) [62]	12.0 (13.7) [71]	10.2 (9.0) [59]	−0.01 (8.4) [59]	−1.1 (−4.7 to 2.3)	0.16	**0.03**	0.52
Triglycerides (mmol/L)	1.5 (0.6) [76]	1.5 (1.1) [62]	−0.1 (0.7) [62]	1.3 (0.8) [71]	1.4 (0.5) [59]	−0.03 (0.6) [59]	0.1 (−0.1 to 0.3)	0.58	0.28	0.43
**Body-composition**										
Weight (kg)	78.8 (15.3) [74]	79.7 (15.6) [63]	−0.28 (3.5) [63]	74.8 (15.3) [71]	74.2 (14.7) [60]	0.57 (2.5) [60]	−0.8 (−1.7 to 0.1)	0.75	0.15	0.19
Fat mass (kg)	24.6 (10.4) [74]	23.7 (10.1) [63]	−1.2^*^ (3.2) [63]	21.3 (8.4) [71]	20.3 (7.3) [60]	−0.2 (2.4) [60]	**−0.9**^#^ (−1.8 to −0.1)	**<0.001**	0.05	0.06
Lean body mass (kg)	54.2 (11.1) [74]	55.9 (12.3) [63]	0.9^*^ (3.3) [63]	53.6 (10.6) [71]	54.0 (11.4) [60]	0.8^*^ (1.9) [60]	0.05 (−0.8 to 0.9)	**<0.001**	0.74	0.92
**Nutrition**										
MEDAS	5.7 (2.2) [72]	6.1 (2.3) [52]	0.40 (2.2) [52]	6.2 (2.2) [63]	6.9 (2.5) [50]	0.5 (2.5) [50]	0.7 (−0.2 to 1.6)	**0.03**	0.09	0.81
**Activity**										
Steps per day^d^	8,069 (3,026) [74]	8,226 (2,687) [68]	−40 (1,680) [67]	8,625 (2,878) [69]	8,121 (2,712) [60]	−475 (2,655) [58]	500 (−175 to 1,176)	0.18	0.48	0.34

mo, months; diff, difference; HbA_1c_, glycosylated hemoglobin.

^a^Sensitive analysis: results of the complete case analysis considering all available data.

^b^Estimates of differences between group changes.

^c^Main effects of mixed-effects models.

^d^Pre = week 1 to week 8 and 6 mo = week 17–week 25.

^*^Significant difference (p <0.05; within groups).

^#^Significant difference (p <0.05; between groups); reference ranges: Hba1c% <5.7; Insulin 20–144 pmol/L; Triglyceride <1.7 mmol/L; Adiponectin & Leptin depends on age and BMI. Significant results are highlighted in bold.

### 3.1. Body composition

After 6 months of intervention, we observed no statistically significant interaction between time and group in body weight [*F*_(2, 247)_ = 1.655, *p* = 0.19], fat mass [*F*_(2, 246)_ = 2.796, *p* = 0.063] and lean body mass [*F*_(2, 196)_ = 0.082, *p* = 0.92] parameters (Table 3). However, body fat mass [*F*_(2, 252)_ = 8.44, *p* < 0.001] revealed a significant time effect and group effect [*F*_(1, 85)_ = 3.966, *p* = 0.05]. Lean body mass also showed a significant time effect [*F*_(2, 202)_ = 13.0, *p* < 0.001; Table 3].

### 3.2. Laboratory parameters

Blood samples failed to reveal a statistically significant interaction effect between time × group for HbA1c% [*F*_(2, 248)_ = 0.450, *p* = 0.64], Insulin [*F*_(2, 241)_ = 1.127, *p* = 0.33], Adiponectin [*F*_(2, 245)_ = 0.144, *p* = 0.87], Leptin [*F*_(2, 243)_ = 0.653, *p* = 0.52] and Triglycerides [*F*_(2, 254)_ = 0.844, *p* = 0.43; Table 3]. Only Adiponectin showed significance in the time effect [*F*_(2, 245)_ = 11.78, *p* < 0.001], and Leptin in the group effect [*F*_(1, 141)_ = 4.85, *p* = 0.03; Table 3].

### 3.3. MEDAS

Statistical analysis of the Mediterranean Diet Adherence Screener (MEDAS) showed no significance in the interaction effect [*F*_(1, 112)_ = 0.056, *p* = 0.81] or in the group effect [*F*_(1, 131)_ = 2.870, *p* = 0.09; Table 3]. We detected a significant increase in mediterranean dietary habit across the groups [*F*_(1, 113)_ = 4.995, *p* = 0.03] during study period (Table 3).

## 4. Discussion

The main findings of this randomized controlled trial involving individualized home-based training and activity feedback information were a reduction in fat mass and an increase in lean body mass, with no differences between patients in IG and CG. The intervention group tended to demonstrate a more reduced fat mass than the control group. Dietary behavior and steps per day did not differ between intervention and control patients. The metabolic laboratory parameters also indicated no group differences, although they were already within the reference range (non-pathological) before the intervention.

### 4.1. Body composition

A reduction in fat mass and increase in lean body mass were evident throughout the entire study group without group differences. We noted a tendency for a greater loss of fat mass in the IG than in the CG [−1.2 vs. −0.2 kg; *p* = 0.06; estimates of differences of group changes after 6 months: −0.9 (−1.8 to −0.1)]. In line with this, we identified no time or group changes in weight. Christensen et al. (21) and Leclerc et al. (44) were unable to demonstrate significant changes in body composition via home- or group-based exercise programs either. However, Christensen's intervention was only 12 weeks and purely home-based interval walking training. Leclerc's intervention period was 12 weeks, during which 1.5 h of cardiovascular and muscular endurance training was done three times per week by supervised groups (21).

Note that the decrease in fat mass and increase in lean body mass should be positively stressed, as sarcopenia promotes the development of cancer and raises the mortality rate of cancer patients (45, 46). The inverse change in fat mass and lean body mass results in an unchanged body weight (Table 3).

A published study showed that the extent of body fat mass-loss depends on the training intensity (47). Courneya et al. (48) came to a similar conclusion when they investigated the influence of different exercise intensities and types in breast cancer patients in different weight categories. The different types of intervention were only aerobic exercise at low or high volume, and a low volume aerobic exercise together with resistance training. Their results showed that normal-weight to slightly overweight patients benefited most from higher-dose exercise, whereas overweight patients benefited most from combined exercise. Our study subjects had a mean BMI of 26.9 (IG) and 25.1 (CG) and the training intensity was the individual heart frequency adapted to each patient's capacity according to their baseline exercise test. Had we taken additional weight-based measurements of exercise intensity, we might have observed a stronger effect on the change in body composition in our study. This assumption is supported by Courneya's results (13), where a recommended increase in physical activity of 10 MET- hours/week among colon cancer patients failed to result in significant weight change. Furthermore, our analysis of the activity data (steps per day) showed no group difference or interaction effect between IG and CG patients. The CG, like the IG, received feedback information on their physical activity, suggesting a relevant effect on activity behavior. In our opinion, this may also be a reason for the lack of group difference in body composition. In addition, the fact that CRBP-TS training was exclusively home-based exercising may be behind the lack of significance. Telemedicine-based exercise interventions in cancer patients have revealed improvements in functional capacity and can maintain such improvement long-term (49), nevertheless, the effects on physical activity have been small (23). Studies involving supervised training in groups have proven to lead to a significant decrease in BMI, body weight, or fat mass (32, 39).

### 4.2. Laboratory parameters

Adiponectin revealed significance in the time effect (*p* < 0.001). We noted an increase in the IG of +1.4 mg/L and in the CG of +1.3 mg/L (estimates of difference between IG vs. CG = 0.2 mg/L) on average. This result is supported by the study by Lee (22), who observed a significant rise in Adiponectin levels after a 12-week exercise intervention entailing increased physical activity of 18 MET/week. The systematic review by Simpson and Singh (50) detected a significant change in Adiponectin levels only in one third of randomized controlled trails.

The Leptin concentrations differed significantly between IG and CG (*p* = 0.03), which is attributable to the IG's higher weight. The change in Leptin concentration did not differ in IG and CG (difference between IG vs. CG = −1.1 ng/mL). The review by Bouassida et al. (51) and studies by Fatouros et al. (52) and Dieli-Conwright et al. (53) also demonstrated such an increase in Adiponectin, and a slight change in Leptin due to aerobic and/or resistance training. However, most of their study subjects presented a higher BMI than ours, and were overweight or obese (52, 53). As mentioned above, our study subjects tended to be of normal weight. Furthermore, their nonsignificant decrease in Leptin could be related to consistent body weight and an only slightly reduced body fat mass (51–54). An intervention's intensity also affects changes in metabolic blood parameters. Thus, a subthreshold training intensity may be responsible for the nonsignificant change in Adiponectin and Leptin levels (47). Sturgeon et al. (47) also failed to demonstrate a drop in Leptin after an exercise intervention, suspecting a correlation with the subthreshold protocol.

As fasting Insulin levels were constant in our IG and CG (−2.3 pmol/L vs. −1.1 pmol/L; *p* = 0.91), we cannot confirm study findings that proved training's positive effect on fasting Insulin in cancer patients (22, 53, 55). All three studies investigated shorter intervention periods than our CRBP study, but more extensive training interventions (i.e., 190 or 210 min/week). A stable body weight or slight change in body composition may also counteract a more marked change in Insulin.

We detected no effect on Triglycerides (difference IG vs. CG = +0.1). Lee et al. (22) reported no significant decrease in Triglyceride levels either after 12 weeks of exercise intervention involving 18–27 MET/week. In contrast, de Jesus Leite et al. (56) reported a significant drop in Triglyceride levels after 12 weeks of resistance training entailing 150 min of exercise per week. Since none of the aforementioned studies also investigated a nutritional intervention, why a significant decrease in Triglyceride levels seems to be documented so seldom is difficult to explain.

### 4.3. MEDAS

Our evaluation of the Mediterranean Diet Adherence Screener (MEDAS) revealed a significant time effect (*p* = 0.03). However, interaction effects and any difference between IG and CG were not evident. The mean of both groups rose by just half a score point, +0.4 in IG and +0.5 in CG. The MD's final adherence can be classified as medium with a score of 6.1 (IG) and 6.9 (CG) (38).

Huo's meta-analysis (30) confirmed the positive impact of MD on body weight and blood parameters. We can thus assume that our CRBP-TS study's missing effects on body weight and laboratory parameters are related to the unchanging MEDAS scores. Note that none of our patients received nutritional instructions, or were encouraged to follow the Mediterranean Diet.

### 4.4. Limitations

Our study obviously has strengths and limitations that need to be understood. One limitation of our multicenter study is that the technologies applied to measure body composition were not the same at all study centers. Furthermore, the training implementation was not monitored on a daily basis—rather, that was sometimes done retrospectively, or after subjects reported having had problems. According to our per-protocol analyses, 74% of the IG fulfilled the recommended 75% of the number of workouts of at least 2.0 per week. To achieve more meaningful results in the future, we will need to monitor home-based training more closely. Thirdly, we can assume that mostly exercise-enthusiastic cancer patients participated in this study revealed by their normal body weight and lean body mass values. This is another potential explanation for their minor improvement in body composition. Fourth, the CG also got activity feedback from their fitness tracker. We therefore assume that there was a motivational influence. Our CG participants may have increased their activity after study entry and thereby limited our ability to detect a difference between groups. We did not conduct complete blinding in our study because that could have triggered a high drop-out rate among CG patients for negative motivational reasons. As another limitation, we must mention the low number of training sessions and low intensity due to the patients' postoperative condition, especially compared to other studies (53, 55, 56).

### 4.5. Conclusion

Individualized home-based training in postoperative cancer patients revealed only small changes, and no group differences in body composition and metabolic laboratory parameters. Activity feedback information given to IG and CG seems to contribute significantly to positive lifestyle management. To the best of our knowledge, the present results are the first describing the effect of home-based training (aerobic and resistance) on the blood parameters Adiponectin, Leptin, fasting Insulin, and Triglyceride concentrations in female and male patients with breast cancer, prostate cancer, or colorectal cancer in conjunction with the acquisition of Mediterranean nutrition. Our study cohort's body composition and laboratory parameters were predominantly in the reference range (non-pathological) at baseline, which may explain the minor change without group differences attributable to the training intervention.

More high-quality studies are needed to explore and demonstrate more accurately the physiological and metabolic changes triggered by exercise in breast, prostate and colorectal cancer patients, and before a true dose–response relationship can be identified.

## Data availability statement

The original contributions presented in the study are included in the article/Supplementary material, further inquiries can be directed to the corresponding author.

## Ethics statement

The studies involving humans were approved by Ethics Committee of the Medical Faculty, University of Leipzig. The studies were conducted in accordance with the local legislation and institutional requirements. The participants provided their written informed consent to participate in this study. Protocol No.: Leipzig 056/20-ek; Dresden BO-EK-581122020; Hannover 9195_BO_K_2020.

## Author contributions

RF and MB conceived and designed the study. RT organized and planned the blood sample analysis. CB, IG, UT, PH, and JV gave advice on the implementation of the study design at the study sites and are involved in recruitment and data acquisition. RF, CB, SD, and JV analyzed and interpreted the data. SD and RF wrote the original draft. All authors substantively revised the work for important intellectual content and have read and approved the submitted manuscript.
