# Supplementary material for: Impact of home-based training and nutritional behavior on body composition and metabolic markers in cancer patients: data from the CRBP-TS study
Source: Front Nutr. 2023 Sep 19;10:1152218. doi: 10.3389/fnut.2023.1152218 (PMC10546323; doi:10.3389/fnut.2023.1152218)
Supplement: Supplementary file 1 [file Data_Sheet_1.docx]

**Table S1:** Laboratory parameters, body composition, nutrition questionnaire and steps per day at baseline and after 6 months (per protocol analysis)

|  | Mean  (SD)  [sample size] | | | |  | | |  | Time effect^c^ | Group effect^c^ | Inter-action effect^c^ |
| --- | --- | --- | --- | --- | --- | --- | --- | --- | --- | --- | --- |
|  | **Intervention group**  **___________________** | | | | **Control group**  **___________________** | | | Difference^b^ 6 months  IG vs. CG (95% CI) | p | p | Group x Time  p |
|  | **pre** | | **6 mo** | **Diff^a^** | **pre** | **6 mo** | **Diff^a^** |  |  |  |  |
| Laboratory |  | |  |  |  |  |  |  |  |  |  |
| Hba1c% | 5.5  (0.4)  [45] | | 5.5  (0.3)  [43] | 0.01  (0.3)  [43] | 5.4  (0.6)  [71] | 5.4  (0.3)  [59] | 0.05  (0.3)  [59] | -0.02  (-0.14 to 0.10) | 0.52 | 0.62 | 0.86 |
| Insulin  (pmol/l) | 63.1  (45.3)  [45] | | 61.77  (29.5)  [43] | -2.6  (34.8)  [43] | 63.9  (53.5)  [71] | 61.8  (38.9)  [59] | -1.1  (49.5)  [59] | -4.2  (-24 to 16) | 0.50 | 0.62 | 0.74 |
| Adiponectin  (mg/l) | 8.7  (6.4)  [45] | | 9.8  (8.0)  [43] | 1.1  (3.5)  [43] | 9.1  (5.5)  [71] | 10.1  (5.8)  [59] | 1.3  (3.3)  [59] | -0.1  (-1.2 to 1.0) | **<0.001** | 0.70 | 0.71 |
| Leptin  (ng/ml) | 16.1  (20.5)  [45] | | 14.3  (15.4)  [43] | -2.5  (11.8)  [43] | 12.0  (13.7)  [71] | 10.2  (9.0)  [59] | -0.01  (8.4)  [59] | -1.4  (-4.9 to 2.2) | **0.04** | 0.10 | 0.74 |
| Triglycerides  (mmol/l) | 1.5  (0.7)  [45] | | 1.6  (1.1)  [43] | 0.05  (0.8)  [43] | 1.3  (0.8)  [71] | 1.4  (0.5)  [59] | -0.03  (0.6)  [59] | 0.1  (-0.2 to 0.3) | 0.24 | 0.19 | 0.83 |
| Body-  composition | |  |  |  |  |  |  |  |  |  |  |
| Weight (kg) | 80.6  (15.0)  [45] | | 80.2  (15.9)  [43] | -0.3  (3.6)  [43] | 74.8  (15.3)  [71] | 74.2  (14.7)  [60] | 0.57  (2.5)  [60] | -0,6  (-1.6 to 0.4) | 0.39 | 0.11 | 0.45 |
| Fat mass  (kg) | 25.1  (11.1)  [45] | | 23.8  (10.2)  [43] | -1.4  (3.2)  [43] | 21.3  (8.4)  [71] | 20.3 (7.3)  [60] | -0.2  (2.4)  [60] | **-1.0***  (-1.9 to -0.1) | **<0.006** | 0.07 | **0.05** |
| Lean body  Mass (kg) | 55.6  (10.8)  [45] | | 56.4  (12.7)  [43] | 1.1  (3.3)  [43] | 53.6  (3.5)  [71] | 54.0  (11.4)  [60] | 0.8  (1.9)  [60] | 0.4  (-0.5 to 1.2) | **<0.001** | 0.49 | 0.34 |
| Nutrition |  | |  |  |  |  |  |  |  |  |  |
| MEDAS | 5.9  (2.3)  [44] | | 6.2  (2.2)  [40] | 0.3  (2.3)  [40] | 6.2  (2.6)  [63] | 6.9  (2.5)  [50] | 0.5  (2.5)  [50] | 0.4  (-1.1 to 0.8) | 0.10 | 0.42 | 0.71 |
| Activity |  | |  |  |  |  |  |  |  |  |  |
| Steps per  day^d^ | 8880  (3037)  [45] | | 8605  (2417)  [45] | -275  (1467)  [45] | 8625  (2878)  [69] | 8121  (2712)  [60] | -475  (2655)  [58] | 176  (-608 to 959) | 0.11 | 0.86 | 0.87 |

Abbreviations: pre = baseline; mo = months; diff = difference; HbA_1c_ = *g*lycosylated hemoglobin; ^a^sensitive analysis: results of the complete case analysis considering all available data; ^b^estimates of differences between group changes; ^c^main effects of mixed-effects models; ^d^pre = week 1 to week 8 and 6 mo = week 17 to week 25; *significant difference (p<0.05; within groups); ^#^significant difference (p<0.05; between groups)

**Table S2** Laboratory parameters, body composition, nutrition questionnaire and steps per day at baseline and after 3 months

|  | Mean (SD) [sample size] | | |  | | | |  |
| --- | --- | --- | --- | --- | --- | --- | --- | --- |
|  | **Intervention group**  **___________________** | | | **Control group**    **___________________** | | | | Difference^b^ 3 months  IG vs. CG (95% CI) |
|  | **pre** | **3 mo** | **Diff^a^** | | **pre** | **3 mo** | **Diff^a^** |  |
| Laboratory |  |  |  | |  |  |  |  |
| Hba1c% | 5.4  (0.4)  [76] | 5.5  (0.4)  [68] | 0.08  (0.3)  [67] | | 5.4  (0.7)  [71] | 5.5  (0.6)  [60] | 0.02  (0.4)  [60] | 0.04  (-0.15 to 0.08) |
| Insulin  (pmol/l) | 68.8  (53.2)  [76] | 63.5  (34.4)  [66] | -1.3  (33.9)  [65] | | 63.9  (53.5)  [71] | 73.2  (85.4)  [60] | 7.9  (74.3)  [60] | 19.6  (-6 to 46) |
| Adiponectin  (mg/l) | 8.4  (5.6)  [76] | 8.8  (6.1)  [66] | 0.6  (3.0)  [65] | | 9.1  (5.5)  [71] | 9.5  (4.9)  [60] | 0.7  (2.6)  [60] | 0.3  (-0.8 to 1.3) |
| Leptin  (ng/ml) | 17.0  (20.3)  [75] | 15.3  (16.9)  [66] | -0.3  (14.5)  [65] | | 12.0  (13.7)  [71] | 9.4  (9.0)  [60] | -2.0  (8.5)  [60] | -2.0  (-5.6 to 1.5) |
| Triglycerides  (mmol/l) | 1.5  (0.6)  [76] | 1.5  (0.8)  [66] | 0.02  (0.5)  [65] | | 1.3  (0.8)  [71] | 1.5  (0.6)  [60] | 0.08  (0.6)  [60] | 0.1  (-0.1 to 0.4) |
| Body-  composition |  |  |  | |  |  |  |  |
| Weight (kg) | 78.8  (15.3)  [74] | 80.8  (14.9)  [68] | -0.005  (3.1)  [67] | | 74.8  (15.3)  [71] | 73.9  (15.3)  [60] | 0.2  (2.1)  [60] | -0,7  (-1.6 to 0.3) |
| Fat mass  (kg) | 24.6  (10.4)  [74] | 23.8  (10.0)  [68] | **-0.9***  (2.6)  [67] | | 21.3  (8.4)  [71] | 19.8  (7.6)  [60] | **-0.8***  (1.9)  [60] | -0.8  (-1.7 to 0.02) |
| Lean body  Mass (kg) | 54.2  (11.1)  [74] | 56.2  (11.6)  [68] | **0.9***  (2.5)  [67] | | 53.6  (10.6)  [71] | 54.1  (11.4)  [60] | **0.9***  (1.7)  [60] | 0.15  (-0.6 to 0.9) |
| Activity |  |  |  | |  |  |  |  |
| Steps per  day^d^ | 8069  (3026)  [74] | 8001  (2800)  [70] | -245  (1630)  [69] | | 8625  (2878)  [69] | 8346  (2499)  [63] | -473  (2002)  [61] | 327  (-351 to 1005) |

Abbreviations: pre = baseline; mo = months; diff = difference; HbA_1c_ = *g*lycosylated hemoglobin; ^a^sensitive analysis: results of the complete case analysis considering all available data; ^b^estimates of differences between group changes; ^c^main effects of mixed-effects models; ^d^pre = week 1 to week 8 and 6 mo = week 17 to week 25; *significant difference (p<0.05; within groups); ^#^significant difference (p<0.05; between groups)
